# Supplementary material for: Comprehensive Annotation and Functional Exploration of MicroRNAs in Lettuce
Source: Front Plant Sci. 2021 Dec 24;12:781836. doi: 10.3389/fpls.2021.781836 (PMC8739914; doi:10.3389/fpls.2021.781836)
Supplement: Supplementary file 2 [file Data_Sheet_8.ZIP › GSM2230749.plot/Lsa-miR159a_Lsat_1_v5_gn_4_92901.1_439_TPlot.pdf]

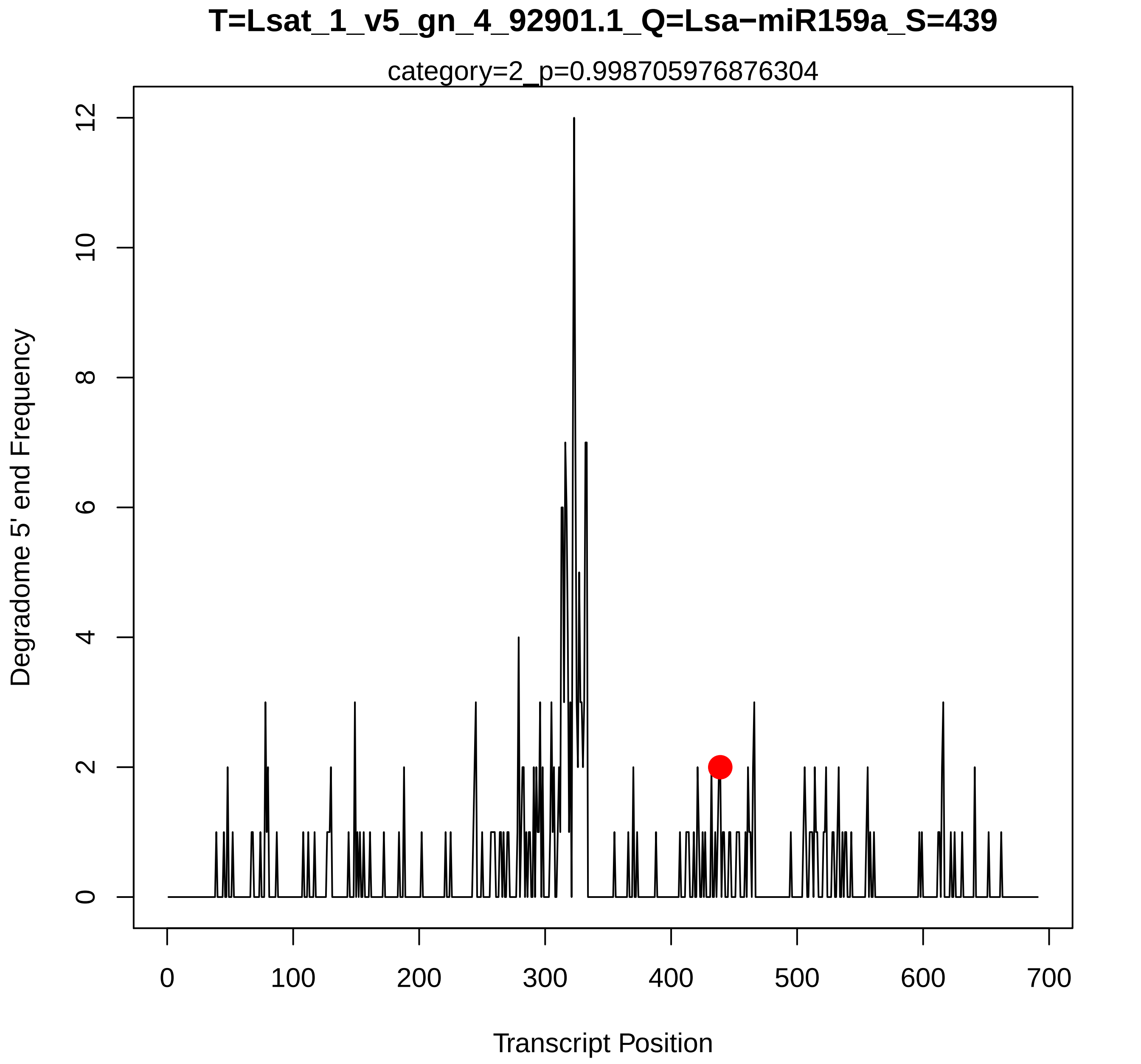
T=Lsat_1_v5_gn_4_92901.1_Q=Lsa−miR159a_S=439
category=2_p=0.998705976876304
Degradome 5' end Frequency
0
2
4
6
8
10
12
0
100
200
300
400
500
600
700
Transcript Position
